# Transcriptomic Analyses of the Biological Effects of Airborne PM2.5 Exposure on Human Bronchial Epithelial Cells

**DOI:** 10.1371/journal.pone.0138267

**Published:** 2015-09-18

**Authors:** Zhixiang Zhou, Yanghua Liu, Fengkui Duan, Mengnan Qin, Fengchang Wu, Wang Sheng, Lixin Yang, Jianguo Liu, Kebin He

**Affiliations:** 1 College of Life Science and Bioengineering, Beijing University of Technology, Beijing, 100124, China; 2 State Key Joint Laboratory of Environment Simulation and Pollution Control, School of Environment, Tsinghua University, Beijing, 100084, China; 3 State Key Laboratory of Environmental Criteria and Risk Assessment, Chinese Research Academy of Environmental Sciences, Beijing, 100012, China; 4 Key Laboratory in Environmental Optics & Technology, Hefei Institutes of Physical Science, Chinese Academy of Sciences, Hefei, 230031, China; Penn State University, UNITED STATES

## Abstract

Epidemiological studies have associated high levels of airborne particulate matter (PM) with increased respiratory diseases. In order to investigate the mechanisms of air pollution-induced lung toxicity in humans, human bronchial epithelial cells (16HBE) were exposed to various concentrations of particles smaller than 2.5 μm (PM2.5) collected from Beijing, China. After observing that PM2.5 decreased cell viability in a dose-dependent manner, we first used Illumina RNA-seq to identify genes and pathways that may contribute to PM2.5-induced toxicity to 16HBE cells. A total of 539 genes, 283 up-regulated and 256 down-regulated, were identified to be significantly differentially expressed after exposure to 25 μg/cm^2^ PM2.5. PM2.5 induced a large number of genes involved in responses to xenobtiotic stimuli, metabolic response, and inflammatory and immune response pathways such as MAPK signaling and cytokine-cytokine receptor interaction, which might contribute to PM2.5-related pulmonary diseases. We then confirmed our RNA-seq results by qPCR and by analysis of IL-6, CYP1A1, and IL-8 protein expression. Finally, ELISA assay demonstrated a significant association between exposure to PM2.5 and secretion of IL-6. This research provides a new insight into the mechanisms underlying PM2.5-induced respiratory diseases in Beijing.

## Introduction

Air pollution is a pervasive environmental health risk factor to individuals from many industrialized societies. Ambient particulate matters (PM), especially particles with an aerodynamic diameter smaller than 2.5 μm (PM2.5), are able to absorb toxic pollutants such as volatile organic compounds (VOCs), heavy metals, and polycyclic aromatic hydrocarbons (PAHs) [1, 2]. It can penetrate deeply into the human respiratory system and reach the blood-air barrier [3]. Long-term exposure to PM2.5 increases the risk of lung cancer and cardiovascular disease [4], while short-term exposure has been associated with various cardiopulmonary diseases such as asthma, bronchitis, arrhythmia, and so on [5]. Although it has been reported that PM2.5-caused oxidative stress and inflammation response are possibly responsible for the different lung diseases [6, 7], the mechanisms underlying PM2.5-induced health effects are still not clear. Moreover, epidemiological evidence indicated that the health effects of PM2.5 correlate with factors such as the population, the location, and the sources of air pollution, [8], which means that different locations exposed to the same level of PM2.5 experience different degrees of toxicity due to different mechanisms.

With the development of the country, the consumption of fossil fuel has increased extensively in China during the last decades, causing an obvious rise in emissions of air pollutants. The air pollution is producing a serious public health problem in China. PM2.5 has become the fourth leading cause of death in China [9]. As China’s capital, Beijing is experiencing severe air contamination. In January 2013, Beijing experienced a heavy air pollution event, and the daily average concentration of PM2.5 in Beijing reached levels as high as 500 μg/m^3^, which is 20-fold higher than the maximum WHO guideline value[10]. It has been reported that airborne PM2.5 has a significant correlation to widespread respiratory irritation symptoms (i.e. Beijing cough) and the increases of outpatient cases [10].

Therefore, it is important to investigate the toxic potential of Beijing airborne particulate matter in order to contribute to a better understanding of the underlying biological mechanisms for PM-induced lung diseases. In this study, we obtained samples of fine particles (PM2.5) from Beijing air in January 2013, and investigated their cytotoxic effects on human bronchial epithelial cells (the 16HBE cell line) by measuring cellular viability and ROS generation. To better understand the possible biological effects associated with PM2.5 exposure, we then obtained global gene expression profiles of cells following acute exposure to PM2.5 using the Illumina RNA-Seq technique, a more precise and sensitive way of characterizing transcriptomes with fewer confounding effects than microarray analysis [11]. Finally, we used quantitative RT-PCR and Western Blot to confirm a selection of the RNA-Seq findings. In this study we show that 16HBE cells exposed to PM2.5 have altered expression of a number of genes involved in responses to xenobtiotic stimuli, metabolic responses, and inflammatory and immune response pathways such as MAPK signaling and cytokine-cytokine receptor interaction. These data provide considerable new information about the adverse health effects of Beijing PM2.5 particles on human bronchial epithelial functions, and also suggest new avenues for further investigation of how ambient PM2.5 affects human pulmonary health.

## Materials and Methods

### 2.1 PM collection

Sampling site was at the roof of the Environmental Lab in Tsinghua University, Beijing, China, a typical Beijing urban sampling site. PM2.5 samples were collected on quartz filters (47 mm, 2 μm, Whatman), the former being used to extract particles for biological investigations, the latter for chemical characterization. Daily PM2.5 was continuously sampled by high-volume samplers (1 m^3^ min^-1^) using filters during Jan. 2013. All of the loaded filters were wrapped in aluminum foil and stored at -20°C until particle extraction.

### 2.2 Particle extraction

To obtain particles for in vitro exposure, quartz filters were soaked with a small amount of 75% alcohol and sonicated for 30 minutes using an ice-cooled water ultrasonic bath sonicator. 5 ml of sterilized water were then added to each group of filters and sonicated three times for 15 minutes each time. The detached particles were dried by lyophilization and suspended in sterilized water to obtain aliquots at a final concentration of 4 mg/mL which were stored at −70°C until further use.

### 2.3 Cell culture and treatments

16HBE cells, an immortalized normal bronchial epithelial cell line, have been characterized and shown to be non-tumorigenic and to retain features of normal bronchial cells [12]. Thus they were used for PM2.5 exposure experiment in this study. The 16HBE cells for use here were purchased from Shanghai SXBIO Biotechnology CO. LTD (Shanghai, China). 16HBE cells were routinely maintained in 5% CO_2_ at 37°C in Roswell Park Memorial Institute (RPMI) 1640 medium (Gibco, USA), supplemented with 10% fetal bovine serum (FBS), 100 U/ml penicillin, and 100 μg/ml streptomycin. For particle exposure, cells were seeded in triplicate 24 hours prior to exposure. Aliquots of PM2.5 suspension was mixed with 1% FBS supplemented medium by sonication for 10 minutes to make the particles evenly distributed, and then applied to the cell culture media. For cell viability assay, 16HBE cells were exposed to PM2.5 at the concentrations of 1.56, 7.81, 15.63, 31.25 and 62.50 μg/cm^2^ (5, 25, 50, 100 and 200 μg/mL) for 24 hours. For enzyme-linked immunosorbent assay (ELISA), PM2.5 was used at the concentrations of 7.81, 15.63, 31.25 and 46.88 μg/cm^2^ (25, 50, 100 and 150 μg/mL). In all other assays, 16HBE cells were treated with PM2.5 at the concentration of 25 μg/cm^2^.

To avoid the potential contribution of the filter leachate to cell toxicity, extracts from clean quartz filters were tested on 16HBE at the beginning of the experiments. The results of the cell viability assay did not reveal significant differences in comparison to the unexposed cells (data not shown).

### 2.4 Cell viability

WST-8 assay [2- (2-Methoxy-4-nitrophenyl)- 3-(4-nitrophenyl)- 5-(2,4-disulfophenyl)- 2H- tetrazolium Sodium Salt] was used to evaluate 16HBE cell viability by using Cell Counting Kit 8 (CCK-8) (Zoman Biotechnology Co., Ltd, Beijing, China) according to the manufacturer instructions. After being exposed to various concentrations of PM2.5 for 24 h, 10 μl of CCK-8 solution was added to each well, and the cells were incubated at 37°C for 4 hours. The absorbance of samples was measured by EnSpire® Multimode Plate Readers (PerkinElmer Inc., MA, USA) at 450 nm. A control with different concentrations of PM2.5 but no cells was used to rule out interference between the particle samples and the detection systems or the assay reagents. The viability of untreated control cells was used to establish the 100% level. The WST-8 tests were replicated three times and the final data were reported as the mean percent (± SD (standard deviation)).

### 2.5 Analysis of intracellular reactive oxygen species (ROS)

Intracellular ROS was measured quantitatively by Cell Observer SD (ZEISS, Oberkochen, Germany) using the ROS-sensitive probe 6-carboxy-2’,7’-dichlorodihydrofluorescein diacetate (carboxy-H2DCFH-DA) (D6883, Sigma, MO, USA). Twenty-four hours after seeding, 16HBE cells were incubated at 37°C with 5 μM DCFH-DA in HBSS solution for 30 minutes, gently washed three times with HBSS and then treated with PM2.5 at a concentration of 25 μg/cm^2^. Changes in intracellular ROS in arbitrary single cells before and after PM2.5 exposure were monitored by the ROS fluorescence intensity at 488 nm excitation wavelength, and the distribution of fluorescence was analyzed using image analysis software AxioVision (ZEISS, Oberkochen, Germany). Three arbitrary single cells were randomly selected in the same field of the microscope in each test and the fluorescence intensity were measured. The experiment was replicated three times.

### 2.6 Gene expression profiles

After 16HBE cells were treated with PM2.5 at a concentration of 25 μg/cm^2^ for 24 hours, total RNA was extracted from control and PM2.5 treated cells using the MirVana kit (AM1560, Ambion, USA) according to manufacturer’s instructions. RNA quality and concentration were measured using Agilent RNA 6000 Nano Reagents kit (Agilent Technologies, USA) by an Agilent 2100 Bioanalyzer (Agilent Technologies, USA) prior to further processing. Extracted total RNA was first treated with DNase I to degrade any possible DNA contamination. Poly-A mRNA was isolated using the oligo(dT) magnetic beads and fragmented into small pieces (about 200 bp). Random hexamer primers were used to synthesize first-strand cDNA by reverse transcription, and RNase H and DNA polymerase I were added to synthesize second-strand cDNA, followed by PCR amplification. After qualitative and quantitative analysis, by Agilent 2100 Bioanalyzer and ABI StepOnePlus Real-Time PCR System respectively, the cDNA libraries were subjected to RNA-sequencing via Illumina HiSeqTM 2000(BGI, Shenzhen, China).

### 2.7 Analysis of differential gene expression

The original image data generated by the sequencer was converted into sequences, which were called raw reads. Data cleaning was used on the full database of raw reads to generate high-quality clean reads. Gene expression profiling was measured by mapping clean reads to assembled sequences using SOAPaligner/SOAP2 [13]. Gene expression levels were calculated by using the RPKM [14] method (Reads Per kb per Million reads), by the following formula: $RPKM=\frac{10^{6}C}{NL/10^{3}}$

Where RPKM is the expression level of a detected gene, C is number of reads that uniquely aligned to the gene, N is total number of reads that uniquely aligned to all genes, and L is number of bases of the detected gene. The RPKM method is able to avoid the influence of different gene lengths and sequencing discrepancies on the calculation of gene expression levels. RPKM values can be directly used for comparing the difference of gene expression among samples, and the ratio of treated sample RPKM to control sample RPKM is represented for each gene as the fold change between the exposed sample and the control. The modified Audic’s [15] method was used to screen for the differentially expressed genes (DEGs) in this study. False discovery rate (FDR) [16] was used to determine the threshold of P value in multiple tests and analysis. We used an FDR of <0.001 and an absolute value of log_2_ ratio ≥1 as the threshold to judge the significance of gene expression difference.

### 2.8 Gene ontology enrichment analysis and functional classification

Gene Ontology (GO), a standardized gene functional classification system, comprises three domains (cellular component, molecular function and biological process), which comprehensively describes properties of genes and their products. We first used enrichment analysis to map all DEGs to GO terms in the database (http://www.geneontology.org/), and calculated gene numbers for every term. Then the hypergeometric test was used to find significantly enriched GO terms in DEGs comparing to the genome background. We used the calculated p-value ≤ 0.05 as a threshold; GO terms that met this condition were defined as significantly enriched in DEGs. GO annotation of DEGs was obtained by Blast2GO program [17] through a search of the NR database. Then WEGO software [18] was used to do GO functional classification for DEGs.

### 2.9 Pathway analysis

Because genes must cooperate with each other to exercise their biological functions, pathway-based analysis helps in further understanding the biological functions of genes. All DEGs were mapped to terms in the Kyoto Encyclopedia of Genes and Genomes (KEGG) [19] database (http://www.genome.jp/kegg/pathway.html), and we looked for significantly enriched metabolic pathways or signal transduction pathways in DEGs using the hypergeometric test. The pathways with Q value of ≤ 0.05 were defined as the significantly changed KEGG pathways.

### 2.10 Quantitative RT-PCR validation

Seven representative genes of DEGs in the RNA-seq data were selected for validation by quantitative reverse-transcriptase PCR (qRT-PCR). All gene specific primers used in this study were designed by Primer Premier 5.0 software and listed in Table 1. Total RNA was extracted from control and PM2.5 treated sample sets using TRIzol Reagent (Ambion, Life technology, USA). Double-stranded cDNA was synthesized from 1 μg total RNA using the PrimeScript RT Reagent Kit with gDNA Eraser (TaKaRa Biotechnology, Dalian, China) according to the manufacturer’s instructions. Quantitative RT-PCR (qRT-PCR) was performed in triplicates using SYBR® Premix DimerEraser (TaKaRa Biotechnology, Dalian, China) with a Stratagene Mx 3000P machine (Agilent, CA, USA). GAPDH was chosen as the housekeeping gene in this study. The mRNA fold changes were calculated using the 2^-△△Ct^ method comparing △Ct of PM2.5 treated cells to △Ct of control untreated samples. Ct values were calculated using MxPro Mx3000P software version 4.01 (Stratagene, Agilent) applying automatic baseline and threshold settings.

**Table 1:** Sequences of primers used in quantitative RT-PCR.

Symbol	Oligonucleotide (5’ to 3’)
**CYP1A1**	sense CCACAGCACAACAAGAGACAC, antisense GAGAAACCGTTCAGGTAGGAA
**TNF**	sense CTGCACTTTGGAGTGATCGG, antisense AACATGGGCTACAGGCTTGT
**IL6**	sense AATGAGGAGACTTGCCTGGTG, antisense GGGTCAGGGGTGGTTATTGC
**IL8**	sense, GGTGCAGTTTTGCCAAGGAG, antisense TTCCTTGGGGTCCAGACAGA
**CCL20**	sense, AGAGTTTGCTCCTGGCTGCT, antisense AAGTTGCTTGCTGCTTCTGATT
**ALOX5AP**	sense ACGAAAGCAGGACCCAGAAT, antisense GTGTAGACCCGCTCAAAGGC
**CXCL3**	sense CAAAGTGTGAATGTAAGGTCCC, antisense CTTCTCTCCTGTCAGTTGGTGC

### 2.11 Western blotting verification

Forty micrograms of total protein from 16HBE cell lysates was mixed with SDS-PAGE loading buffer and then boiled for 5 minutes. Proteins in the cell lysates were separated in 10% sodium dodecyl sulfate polyacrylamide gels and electrotransferred to polyvinylidene difluoride (PVDF) membranes (Amersham Biosciences, Sweden), with the nonspecific binding sites blocked by 5% non-fat milk in 0.01 mol/L PBS, pH 7.4, for 2 hours at room temperature. Membranes were then incubated with rabbit polyclonal antibodies to GAPDH (Bioworld, MN, USA), CYP1A1, IL-6 and IL-8 (Bioworld, MN, USA) for 2 hours at room temperature. Membranes were washed 3 times with PBST for 10 minutes each, and then further incubated with RDye800® conjugated Goat anti-rabbit IgG (1:10,000 dilution) (KPL, MN, USA) for 45 minutes at room temperature. After three 10-minute rinses, the blots were visualized using the Odyssey infrared imaging system (LI-COR Biosciences, CA, USA). This generated semiquantitative data that reflected the differences in protein expression between control untreated and PM2.5 treated samples.

### 2.12 Cytokine release

After 5 or 24 hours of treatment, cell culture supernatants were collected from 16HBE cells, centrifuged for 10 minutes at 1000rpm to remove debris, and stored at -70°C until use. DuoSet ELISA development systems (R&D Systems, MN, USA) were used to measure cytokine proteins (TNF-α, IL-6, and IL-8) in culture supernatants. Each assay was performed following the manufacturer’s instructions and measured in a Multimode Plate Reader (PerkinElmer Inc., MA, USA) at 450 nm.

### 2.13 Statistical analysis

All data were expressed as mean ± S.D., for comparisons of the means, we used Student t-test. P values less than 0.05 were considered statistically significant.

## Results

### 3.1 Exposure to PM2.5 from Beijing reduces the viability of bronchial epithelial cells

**Fig 1:**
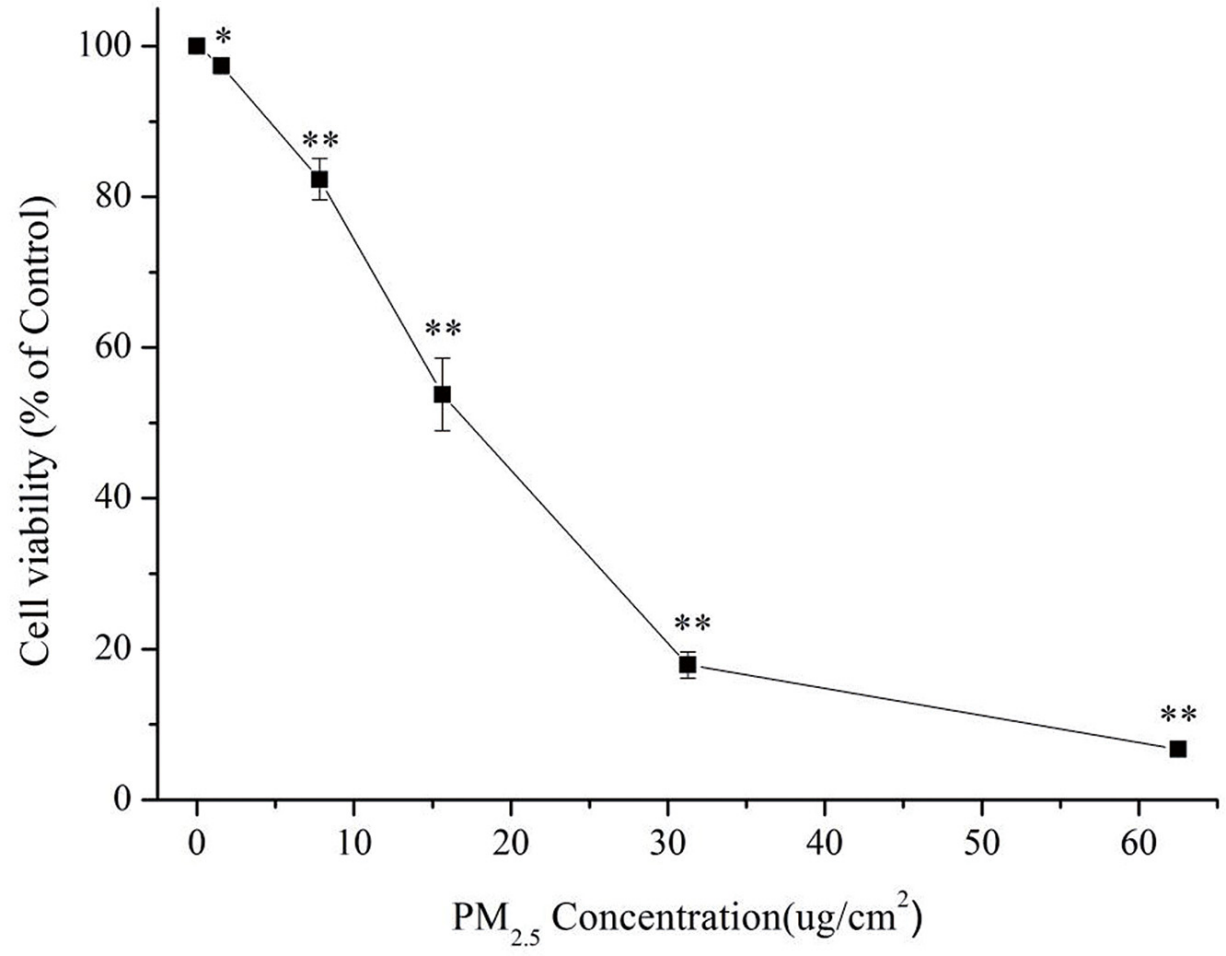
The effect of PM2.5 concentrations on cell viability in 16HBE cells. Cells were exposed to various concentrations of PM2.5 (1.56, 7.81, 15.63, 31.25 and 62.50 μg/cm^2^ (5, 25, 50, 100 and 200 μg/mL) for 24 hours. Cell viability was expressed as a percentage of unexposed control (mean ± S.D.). (*) indicates significant difference (p < 0.05) when compared to control, (**) represents significant difference (p < 0.01) compared to control.

As an initial investigation of the potential toxicity of PM2.5 extracted from Beijing air samples, we incubated 16HBE cells with various concentrations of PM2.5 and measured viability by CCK8 assay after 24 hours of exposure. A progressive decrease in cell viability was observed with increasing concentrations of PM2.5 (Fig 1). Significant differences compared with controls were observed, starting at a PM2.5 concentration of 1.56 μg/cm^2^ (5 μg/mL) and increasing dose-dependently up to 62.50 μg/cm^2^. The IC50 value for PM2.5 to reduce 16HBE cell viability by 50% was 15.63 μg/cm^2^ (50 μg/mL), and the IC70 value was 25 μg/cm^2^ (80 μg/mL).

### 3.2 Exposure to PM2.5 rapidly enhances production of reactive oxygen species

**Fig 2:**
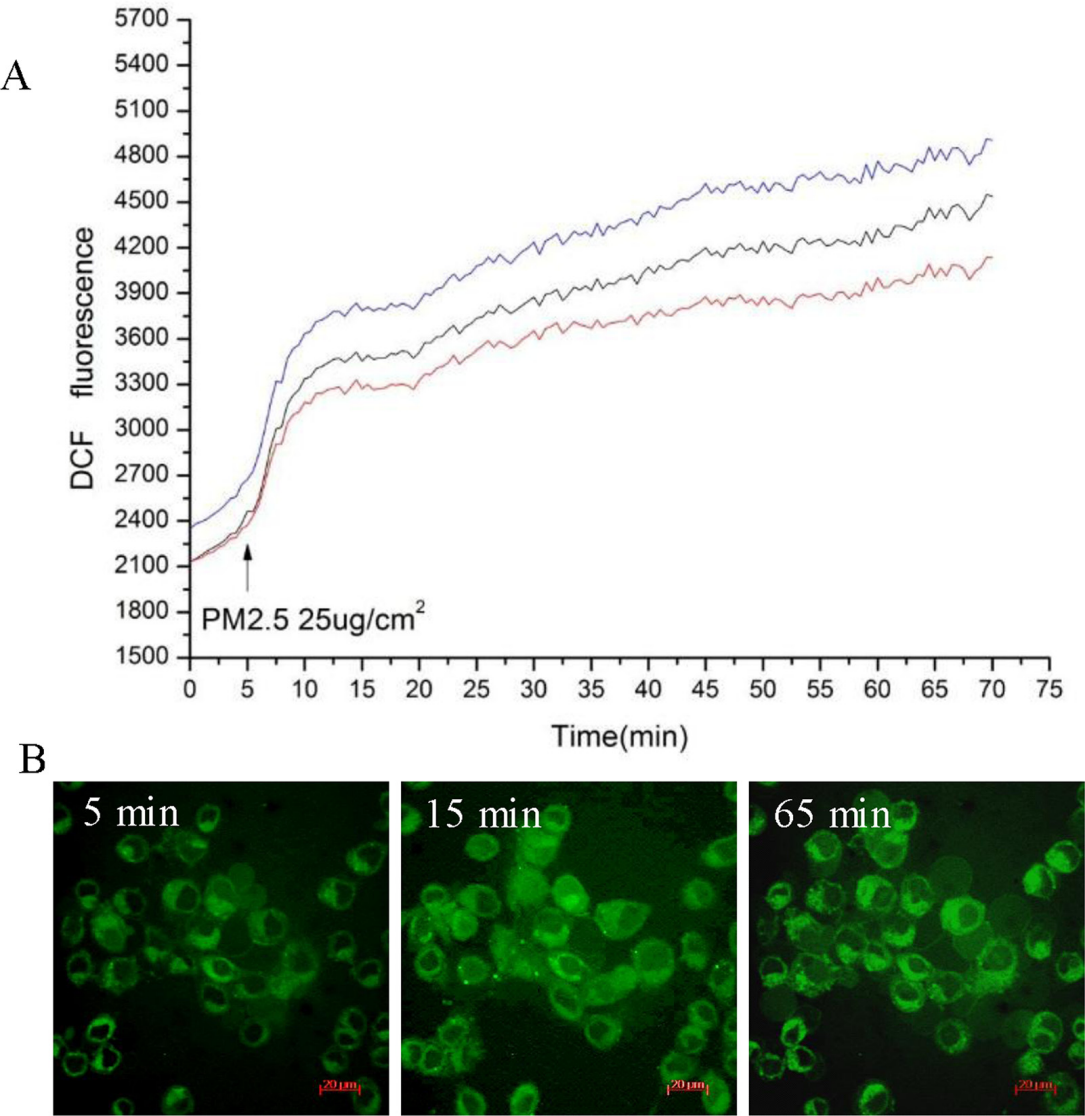
PM2.5 induces ROS generation in 16HBE cells. The cells were incubated with 5 μM DCFH-DA and then treated with PM2.5 at the concentrations of 25 μg/cm^2^. ROS production was monitored with the fluorescence intensity by microscopic analysis. A. Changes in intracellular ROS levels before and after PM2.5 exposure. Time 0 means the start of a measurement. The arrow indicates the time at which PM2.5 at the concentration of 25 μg/cm^2^ was added to culture media. The three lines show the DCF fluorescence intensity obtained from three arbitrary single cells in the same field of the microscope. B. Fluorescent localization of ROS. The time labeled on the time series photographs corresponds to the time point in Fig 2A. While the first image was recorded just before the start of PM2.5 exposure (as control), the second and third ones were recorded at 10 and 60 minutes after exposure. Scale bar in graphs, 20 μm.

To investigate the possible changes in intracellular redox homeostasis, a measurement of oxidative stress, ROS generation within HBE cells after PM2.5 treatment was measured by fluorescence microscopy. Intracellular ROS was assessed using the probe carboxy-H2DCFH-DA, which produces green fluorescence when it enters a cell, reacts with ROS and loses its acetate groups. As shown in Fig 2, fluorescence intensity was significantly increased in cells after exposure to PM2.5 (25 μg/cm^2^), while no significant change in cells treated with extracts from clean quartz filters (S1 Fig). This indicates that exposure to PM causes intracellular ROS generation.

### 3.3 Exposure to PM2.5 significantly alters expression of inflammatory and other genes

**Fig 3:**
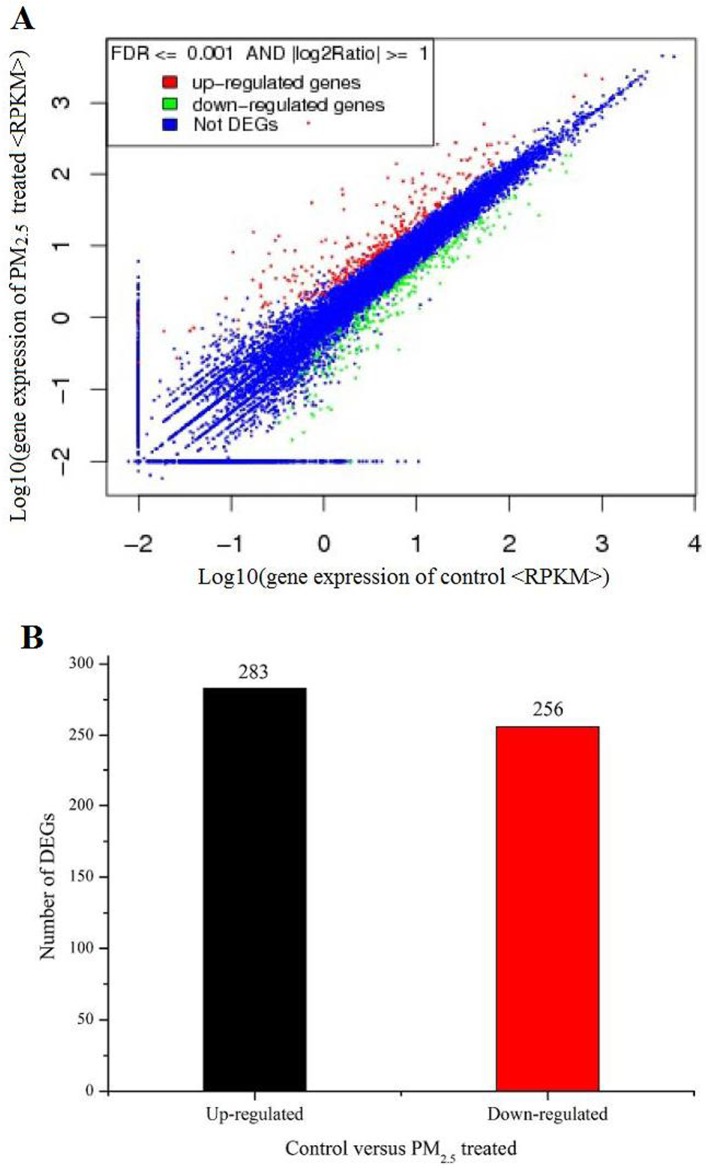
Analysis of differentially expressed genes between PM2.5 treated and untreated samples. A. Gene expression level of control versus PM2.5 treated. FDR<0.001 and the absolute value of log2 fold change ≥ 1 were used as the threshold to judge the significance of gene expression difference. B. Summary of the numbers of differentially expressed genes in the PM2.5 treated sample versus untreated control.

To learn about the molecular pathways up-regulated and down-regulated by exposure to air pollution particulate matter, we performed whole-genome expression profiling using RNA-Seq data from PM2.5 exposed 16HBE cells and unexposed controls. Analyzing this data revealed that PM2.5 had a highly significant and extensive effect on gene expression in 16HBE cells. In total, 539 genes were identified as differentially expressed genes (DEGs), defined as genes with FDR<0.001 and the absolute value of log2 fold change ≥ 1 in a comparison of PM2.5 treated and untreated cells. Among all the DEGs, 283 genes were up-regulated and 256 genes were down-regulated (Fig 3). Genes involved in the response to xenobiotic stimuli, inflammatory response and immune response were among the top 10 differentially regulated genes in PM2.5 treated 16HBE cells compared to untreated control cells (Tables 2 and 3).

**Table 2:** Top 10 up-regulated genes in PM2.5 treated 16HBE cells.

Gene	Length	log2(FC)	Description
CYP1A1	2608	9.54	Cytochrome P450, Family 1, Subfamily A, Polypeptide 1
INHBA	2175	6.88	inhibin beta A chain precursor
TNF	1669	6.57	tumor necrosis factor
SERPINB2	2180	6.46	plasminogen activator inhibitor 2 precursor
IL6	1201	6.28	interleukin-6 precursor
IL8	1718	5.75	interleukin 8
CXCL3	1166	5.39	C-X-C motif chemokine 3
CCL20	851	5.31	C-C motif chemokine 20 isoform 1
TNFAIP3	4446	5.29	tumor necrosis factor alpha-induced protein 3
KIAA1644	6741	5.11	Uncharacterized Protein

**Table 3:** Top 10 down-regulated genes in PM2.5 treated 16HBE cells.

Gene	Length	log2 FC	Description
ALOX5AP	1242	-7.60	five-lipoxygenase activating protein (FLAP)
TENM2	9645	-4.58	Teneurin Transmembrane Protein 2
RERG	2325	-4.52	ras-related and estrogen-regulated growth inhibitor isoform 2
SEMA6D	6109	-4.52	semaphorin-6D isoform 5 precursor
IVL	2165	-4.30	Involucrin
PSG4	2059	-4.19	pregnancy-specific beta-1-glycoprotein 4 isoform 2
MPPED2	5693	-4.19	chromosome 11 open reading frame 8 variant
MAF	6887	-4.12	transcription factor Maf
DPCR1	5306	-4.01	diffuse panbronchiolitis critical region 1 protein
FILIP1L	4211	-3.93	Filamin A Interacting Protein 1-Like

**Fig 4:**
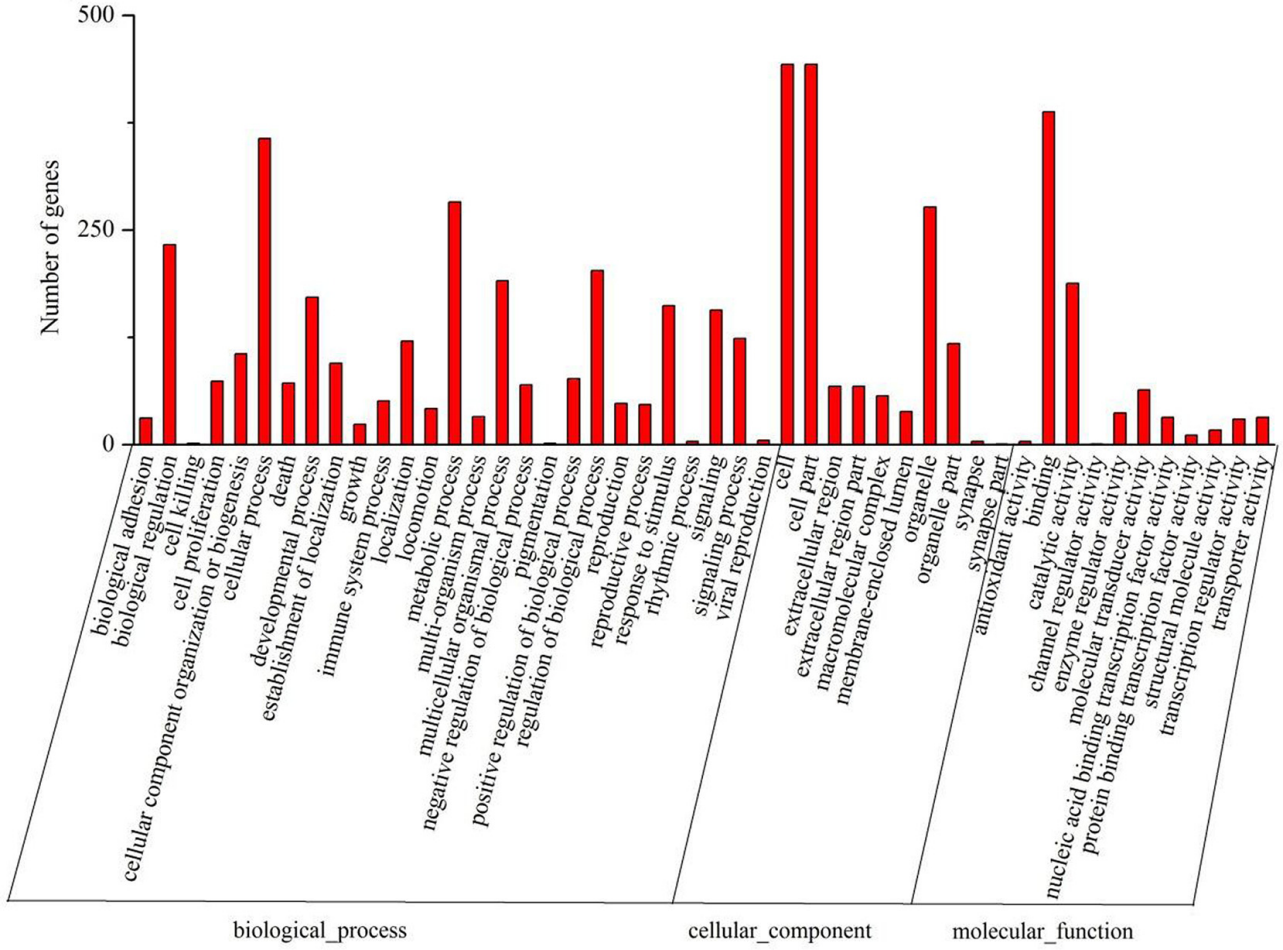
Gene Ontology (GO) classification of genes differentially expressed between PM2.5 treated and untreated samples. The functions of genes identified cover three main categories: biological process, cellular component, and molecular function. The left and right y axes indicate the percentage and the number of genes in a category respectively.

**Fig 5:**
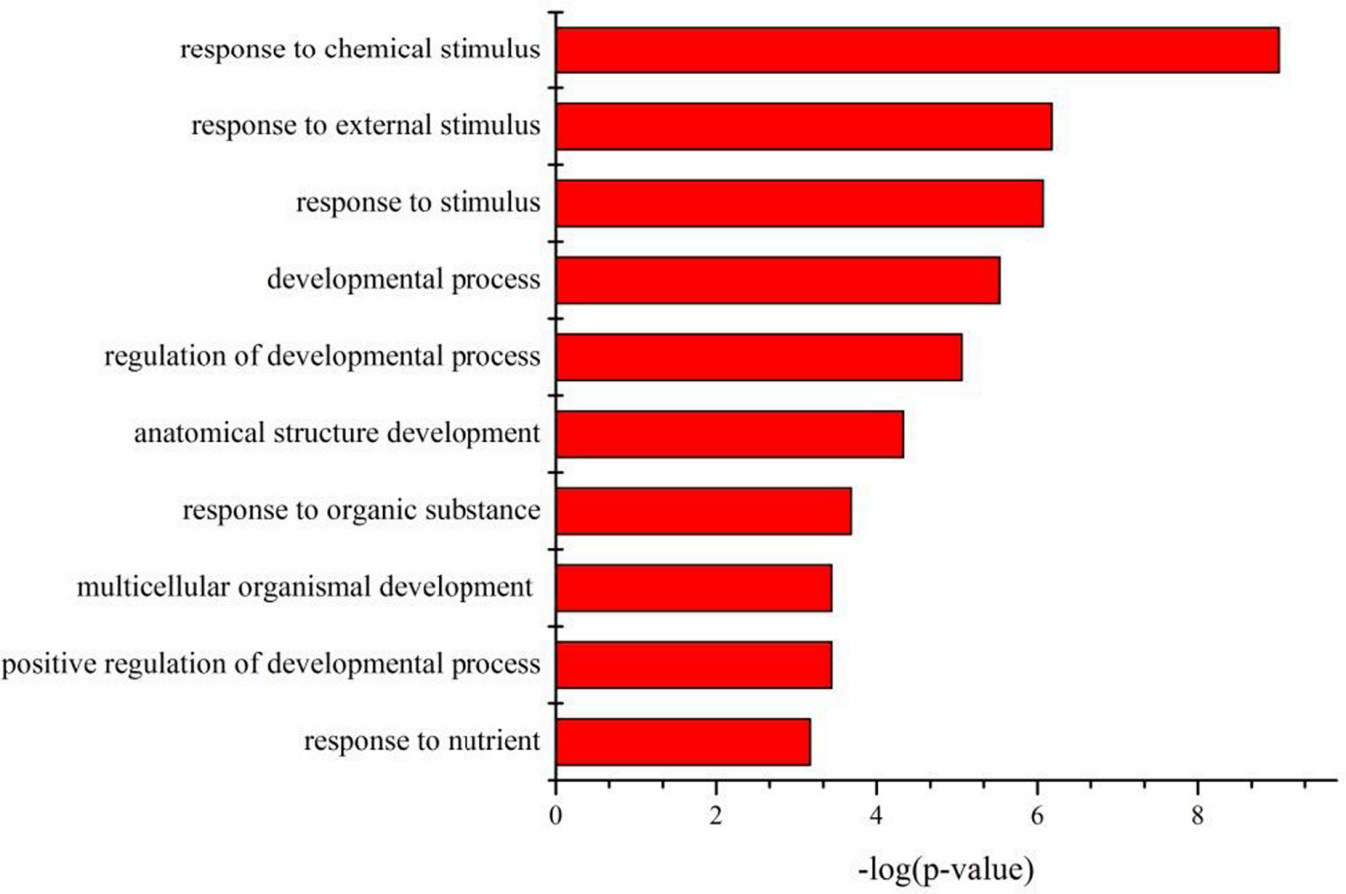
The top 10 biological processes identified by GO analysis of differentially expressed genes. Bars represent-log(P-value). P-value was calculated by hypergeometric test.

We subjected all the DEGs to analysis for relationships as revealed by Gene Ontology (GO) category and Kyoto Encyclopedia of Genes and Genomes (KEGG) pathway. As shown in Fig 4, binding and catalytic activity are among the most represented molecular function categories. As for biological process, the top 10 GO categories related to response to chemical and other stimuli, regulation of developmental processes, and response to nutrients, as shown in Fig 5. Most of these significantly affected GO terms seem to be involved in the response to PM2.5 stimulation, suggesting that cells adjust their metabolism considerably to the presence of PM2.5 in their immediate environment.

Seven KEGG pathways were found to be significantly changed with a Q value of ≤ 0.05 calculated by hypergeometric test (Table 4). Overall, this pathway analysis demonstrates a cellular response centered along the axis of inflammatory/immune processes.

**Table 4:** The significant pathways for the DEGs. The arrows ↑ and ↓ indicate up- or down-regulation.

KEGG pathway	Count	P-value	Q-value	Genes
Rheumatoid arthritis	15	4.97E-07	1.07E-04	TNF↑,IL6↑, IL8↑,CXCL3↑,CCL20↑,CXCL1↑,CXCL2↑,IL1A↑,JUN↑, IL1B↑, ICAM1↑, ANGPT1↓, TGFB2↓,TNFSF13↓, CTSL2↓
Amoebiasis	22	1.22E-06	1.31E-04	TNF↑,SERPINB2↑, IL6↑, IL8↑,CXCL3↑, CXCL1↑,CXCL2↑, NAV3↑, IL1B↑, SERPINB3↑, SERPINB4↑, SERPINB8↑, ZNF469↑,SERPINB9↑, MEGF9↑, DPCR1↓, NAV2↓, SYNPO↓, COL5A1↓, AHNAK↓, TGFB2↓, SYT1↓
NF-kappa B signaling pathway	15	3.18E-04	2.28E-02	TNF↑, IL8↑,CXCL3↑, TNFAIP3↑, CXCL1↑, CXCL2↑, PTGS2↑, IL1B↑,NFKBIA↑, RELB↑, ICAM1↑, BIRC3↑,SYNGR3↓,CARD14↓, SYT1↓
Cytokine-cytokine receptor interaction	20	5.79E-04	3.11E-02	INHBA↑, TNF↑,IL6↑, IL8↑, CXCL3↑,CCL20↑,CXCL1↑,IL24↑, CXCL2↑,IL1A↑,LIF↑, IL1B↑,TNFRSF21↑,RELT↑,GDF6↓, TGFB2↓,NGFR↓, TNFSF10↓, TNFSF13↓, LIFR↓
Metabolism of xenobiotics by cytochrome P450	9	1.12E-03	4.19E-02	CYP1A1↑,AKR1C1↑, CYP1B1↑,AKR1C2↑, AKR1C3↑, ALDH3A1↑,ALDH1A3↑,CBR3↑, ALOX5AP↓
MAPK signaling pathway	24	1.17E-03	4.19E-02	TNF↑, DUSP6↑, NAV3↑, IL1A↑, JUN↑, IL1B↑, DUSP5↑, DUSP4↑,RELB↑,RAP1GAP↑,NR4A1↑,CDC25B↑,FGF1↓, NAV2↓, DUSP10↓, CACNG4↓, PPM1N↓, SYNGR3↓,PTPRR↓, TGFB2↓,SYT1↓, FGFR2↓, NFATC4↓, MAP3K12↓
Fructose and mannose metabolism	7	1.42E-03	4.35E-02	AKR1C1↑, AKR1C2↑,AKR1B10↑, AKR1C3↑, HKDC1↑,ALDOC↓, MTMR10↓

### 3.4 Up-regulation of genes after PM2.5 exposure can be robustly detected by both RNA-seq and quantitative reverse-transcriptase PCR

**Fig 6:**
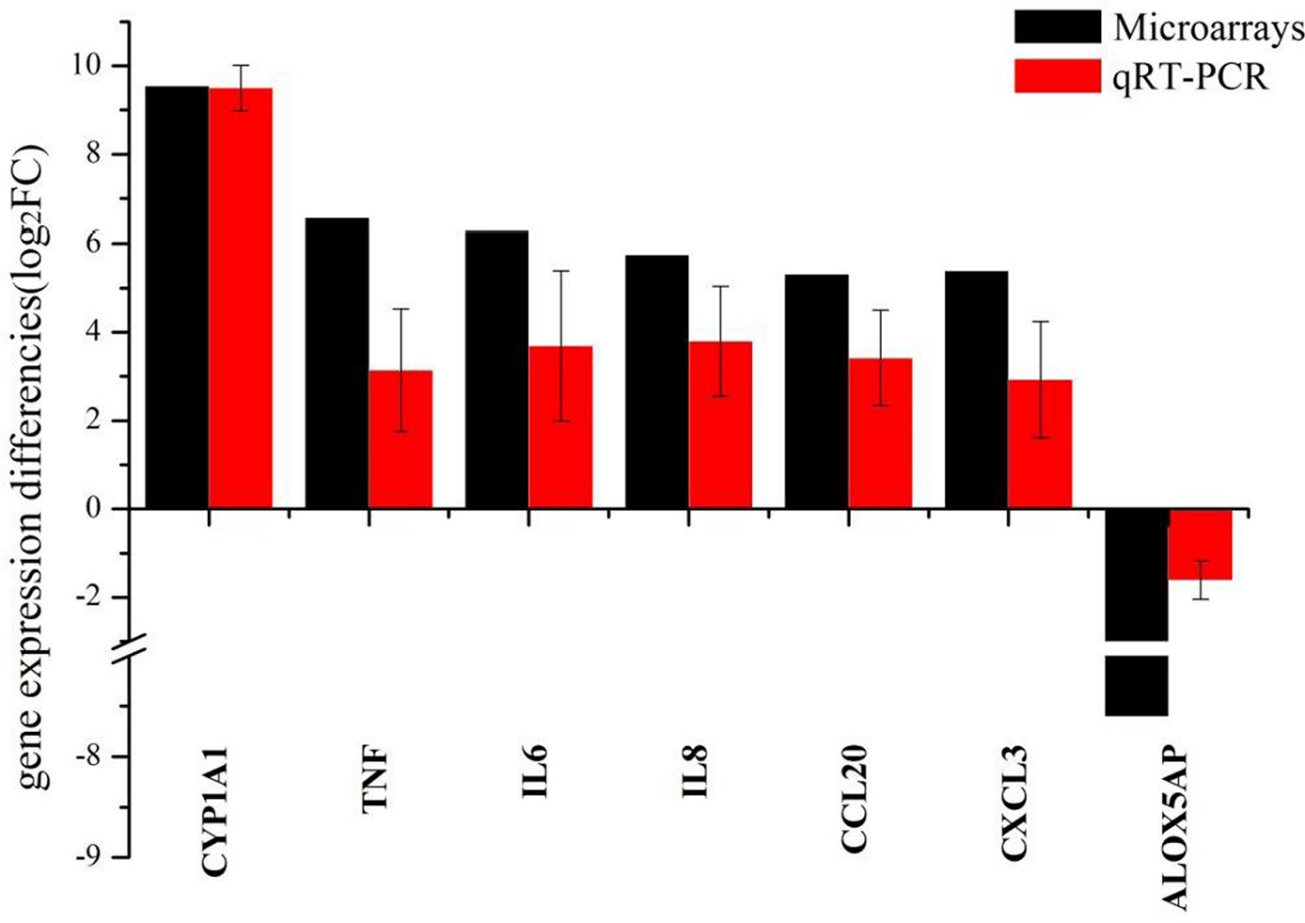
Correlation between RNA-seq data and qRT-PCR data. The fold changes were calculated using the 2^-△△Ct^ method comparing PM2.5 treated cell to control untreated samples. All Ct values were normalized to GAPDH.

To validate the RNA-seq results in this study, we selected seven DEGs of particular relevance to pulmonary inflammation and sought to corroborate the RNA-seq data usingquantitative RT-PCR. Analyzing qRT-PCR data indicated that these seven genes were indeed differentially expressed, following a similar pattern to the RNA-seq data (Fig 6). However, for 6 of the 7 genes, the RNA-seq data showed a more significant difference between PM2.5 treated and untreated cells than was seen in the qRT-PCR results, which may be caused by the lower sensitivity of qRT-PCR than of RNA-seq. Overall, the good correlation between RNA-seq and qRT-PCR data strongly confirmed that RNA-seq results are reliable in this study.

### 3.5 Selected RNA transcipts up-regulated by PM2.5 are also up-regulated at the protein level

**Fig 7:**
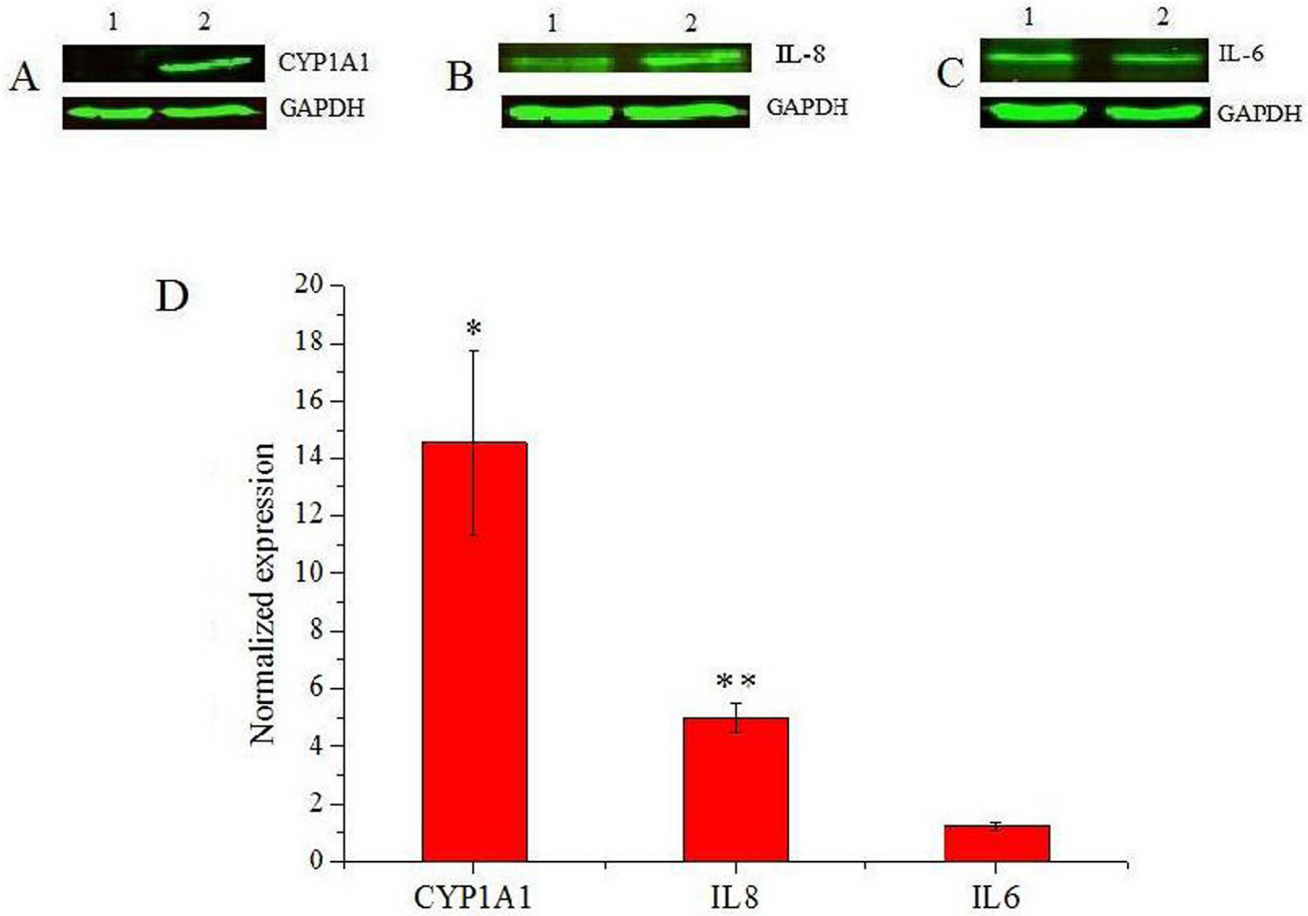
The fold changes in CYP1A1, IL-8 and IL-6 expression between control and PM2.5 treated 16HBE cells confirmed by western blot analysis. A-C. The expression of CYP1A1, IL-8 and IL-6, respectively, detected by western blot analysis of cell lysates. In each gel image the left lane (1) contained untreated control cells and the right lane (2) contained cells treated with 25 μg/cm^2^ PM2.5. GAPDH was used as a loading control. D. Quantitation of western blot band intensity (n = 3). Data is expressed as mean ± SD. (*) indicates significant difference (p < 0.05) when compared to control, (**) represents significant difference (p < 0.01) compared to control.

**Fig 8:**
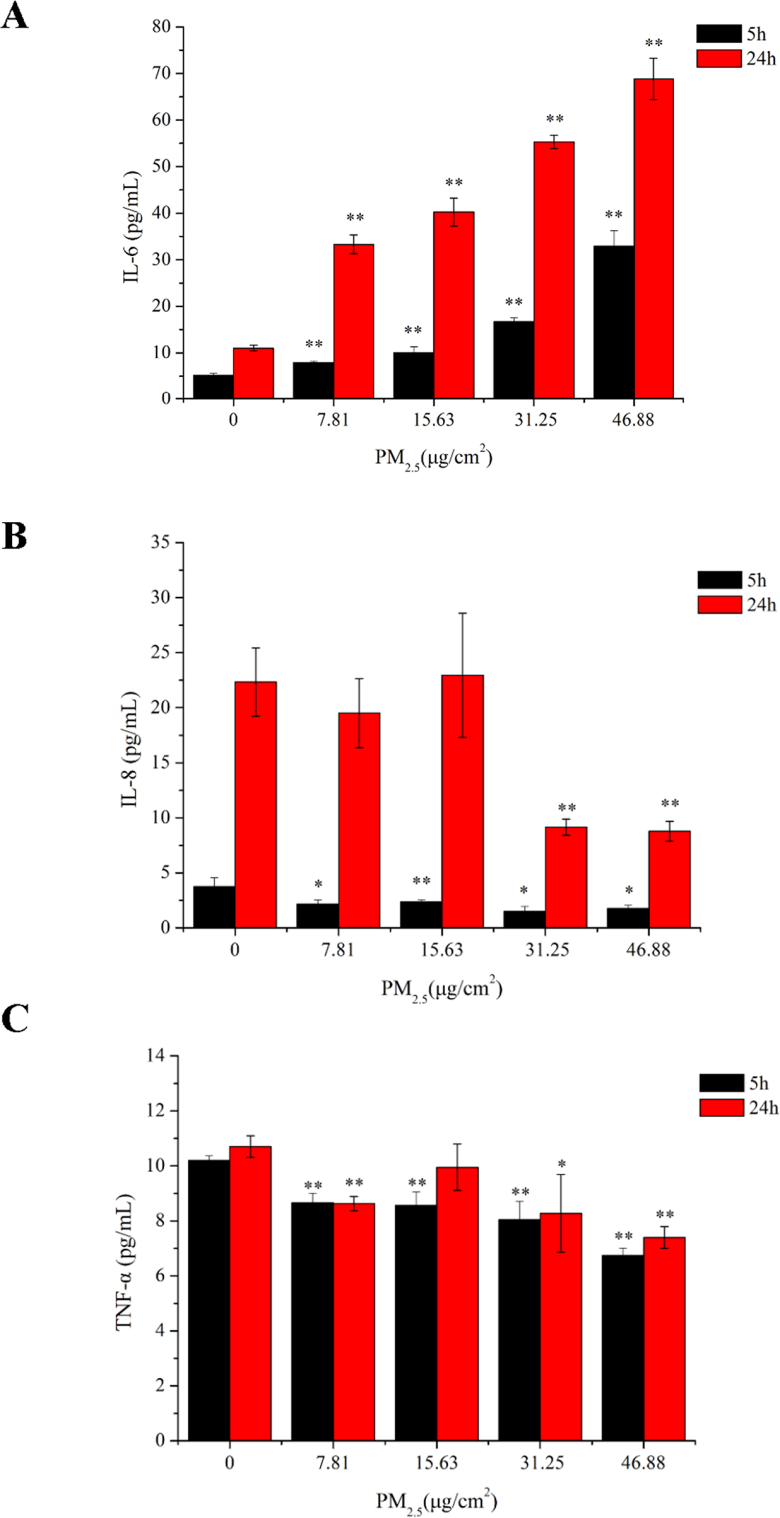
Cytokine release in 16HBE cells exposed to various concentrations of PM2.5. 16HBE cells were exposed to PM2.5 for 5 hours and 24 hours, whereafter the culture supernatants were collected and assessed for (A) IL-6, (B) IL-8 and (C) TNF-α. The data are mean ± SD of independent experiments (n = 3). (*) indicates significant difference (p < 0.05) when compared to control cells (exposed to 0 μg/cm^2^ PM2.5 over the same period), (**) represents significant difference (p < 0.01) compared to control.

We further investigated the effect of PM2.5 on the phenotype of bronchial epithelial cells by using western blot to measure protein expression of CYP1A1, IL-8 and IL-6. These three genes were selected for protein expression analysis because of their important roles in cell processes or pathways, including response to xenobiotic stimuli (CYP1A1) and inflammatory or immune response (IL-8 and IL-6). The results showed that CYP1A1 was significantly up-regulated in PM2.5 treated 16HBE cells compared with untreated controls (Fig 7A and 7D). Similarly, PM2.5 induced a significantly higher IL-8 synthesis than in controls (Fig 7B and 7D). Although we did not see increased protein levels of IL-6 in lysates of PM2.5 treated cells (Fig 7C and 7D), we saw increased IL-6 release in the culture medium (Fig 8). Therefore, this data on protein expression strongly supports the results obtained by RNA-seq in PM2.5 treated 16HBE cells.

### 3.6 Secretion of IL-6 was induced by PM2.5 in a dose-dependent fashion

To investigate the proinflammatory potential of PM2.5, we analyzed the release of cytokines by measuring IL-6, IL-8 and TNF-α levels in cell supernatants. 16HBE cells were exposed to PM2.5 at various concentrations, and at 5 and 24 hour timepoints cytokine production was measured using ELISA. The results (Fig 8A) show that 16HBE cells spontaneously release a low level of IL-6, which is significantly enhanced by all four concentrations of PM2.5 at both 5 hours and 24 hours. Moreover, the release of IL-6 in response to PM2.5 was dose dependent at both timepoints, and at each concentration of PM2.5 the IL-6 level at 24 hours was significantly higher than the level at 5 hours. The release of IL-8 (Fig 8B) was much higher at 24 hours in comparison with 5 hours at all concentrations and PM2.5 exposed cells had significantly lower levels of IL-8 in the culture supernatant, an effect that was particularly noticeable at 24 hours. Finally, when we measured TNF-α, 16HBE cells consistently produced similar levels at both timepoints, with all values detected as between 6 and 12 pg/ml; by comparison, both IL-6 and IL-8 showed a much greater dynamic range, with the highest 24-hour values over tenfold higher than the lowest 5-hour readings. However, within this limited range of protein levels, there was actually a slight decrease in TNF-α at both 5 and 24 hour timepoints, though this effect was not reliably dose-dependent (Fig 8C).

## Discussion

Beijing, the capital city of China, has been suffering from heavy haze events in recent years, especially during January 2013 [10, 20, 21]. PM2.5, as the major air pollutant during the heavy haze days, received an extensive attention due to its impact on human health. This study aimed to use 16HBE cells as a model of target tissue for inhalation exposure, to identify biological processes involved in the toxic effects of PM2.5 collected in January 2013 in Beijing, China.

Here we have shown that Beijing’s PM2.5 leads to concentration-dependent cytotoxicity as assessed through survival rate analysis using the CCK-8 method in 16HBE cells. PM2.5 caused 50% lethality (LC50) at about 15.63 μg/cm^2^ and 70% lethality (LC70) at about 25 μg/cm^2^ after 24 hours of incubation. PM2.5 also stimulated generation of reactive oxygen species (ROS) within the first 10 minutes of exposure in cells. ROS can initiate cellular stress and result in release of transcription factors, inflammatory mediators and activation of kinase cascades, ultimately leading to cell injury [22, 23]. Therefore, to identify biological processes and pathways involved in the toxic effects of Beijing’s PM2.5, RNA-Seq was carried out to decipher the entire transcriptome of the bronchial epithelial cell line after exposure to a complex mixture of PM2.5.

Our analysis of differentially expressed genes in the present study shows that many genes involved in response to oxidative stress were significantly induced by Beijing’s PM2.5, such as HMOX1, GPX2, NQO1, SMOX and SOD2. HMOX1, the inducible isoform of heme oxygenase (HO-1), is a typical oxidative stress marker [24, 25].The significant up-regulation of HMOX1 indicates the presence of oxidative stress responses in 16HBE cells exposed to PM2.5. Diesel particles have been reported to increase oxidative stress in endothelial tissue and induce the production of HMOX-1 [26]. Oxidative stress can be initiated by the formation of reactive oxygen species (ROS) within affected cells. The induction of ROS by PM2.5 observed in the present study further substantiates the generation of oxidative stress. PM-induced ROS formation has been widely reported and is related to the presence of soluble metals as well as PAHs in ambient PM [27, 28].

ROS production is promoted by PAHs usually through the activation of cytochrome P-450, as previously demonstrated in BEAS-2B cells [29]. The pathway analysis in the present study also indicates that the metabolism of xenobiotics by cytochrome P450 is significantly perturbed by PM2.5 in Beijing which is known to have a high quantity of PAHs [30]. The xenobiotic response genes CYP1A1 and CYP1B1 in this perturbed pathway were both up-regulated by PM2.5, and CYP1A1 was shown to be significantly increased at the protein level as well. CYP1A1, a member of the cytochrome P450 family, is a phase I enzyme in the xenobiotic metabolizing pathway and can be stimulated by PAHs [31–33]. Consistently with our data, a similar pattern of gene expression for both CYP1A1 and CYP1B1 induced by PM2.5 with high concentrations of PAHs in five Chinese cities further confirms the importance of PAHs in triggering expression of xenobiotic response genes [30]. In addition, NQO1, a metabolizing enzyme of the xenobiotic metabolizing pathway responsible for detoxification of numerous foreign compounds, is often associated with ROS production that then activates antioxidant responses [34].

As previously reported for PM, PAHs trigger the generation of ROS which may promote oxidative injury and inflammatory response through activation of the transcription factor NF-kB [35]. Correspondingly, our investigation showed that the NF-kB signaling pathway was significantly changed in 16HBE cells exposed to PM2.5. Moreover, it has been reported that primary culture HBE cells respond to ambient PM treatment by up-regulation of CCL20, most likely through activation of MAPK-related pathways [36]. Interestingly, our study showed similar results, with the MAPK signaling pathway significantly perturbed by PM2.5 along with up-regulation of CCL20. Furthermore, it has been demonstrated that activation of MAPK signaling pathway likely plays an important role in the PM2.5-induced production of several inflammatory cytokines [37].

In our study, PM2.5 caused down-regulated genes such as ALOX5AP and DPCR1 in the RNA-seq analysis. ALOX5AP plays a role in the 5-lipoxygenase (LO) pathway, which is known causative factors of asthma, allergy and atopy[38]. DPCR1 was reported as a genetic marker for diffuse panbronchiolitis[39]. It means that inflammatory responses play an important role in adverse respiratory outcomes. Numerous toxicological studies have revealed the relationship between PM and release of cytokines by lung cells [32, 36]. In this study, PM2.5 consistently up-regulated a number of inflammatory response genes including robust expression of INHBA, TNF-α, IL6, IL8, CXCL3, CCL20, CXCL1, IL24, CXCL2, IL1A and IL1B in the RNA-seq analysis. In addition, qRT-PCR experiments verified that PM2.5 significantly up-regulate gene expression of TNF-α, IL6 and IL8. However, no significant change in TNF-α protein secretion was observed in culture supernatants of 16HBE cells, whatever the concentrations of PM2.5 and the exposure duration. Similar results have been reported in BEAS-2B cells with respect to TNF-α protein secretion [31]. Concerning IL-6 protein secretion, we saw a highly significant PM2.5 dose-dependent and time-dependent effect on IL-6 levels in the culture supernatants of 16HBE cells. Meanwhile, other researchers have shown a similar pattern of IL-6 secretion in BEAS-2B, A549, Calu-3 and RAW 264.7 cells exposed to PMs [30, 40–42]. Although we saw no effect of PM2.5 on intracellular IL-6 protein expression as measured by Western blot analysis, our observation of enhanced extracellular IL-6 secretion after PM2.5 exposure agrees with the studies described above. IL-6 is known to be an excellent marker for the pro-inflammatory response and has been utilized in models wherein IL-6 expression in vitro accurately predicts IL-6 expression in vivo. Moreover, Nazariah SS et al. revealed that PM was one of the factors contributing to an increased level of IL-6 measured in sputum samples, and thus IL-6 can be suggested as a biomarker for respiratory problems in healthy children [43].

In conclusion, we have identified hundreds of genes differentially regulated by PM2.5 through our use of RNA-Seq analysis, the next generation transcriptomics technology that shows a greater dynamic range than microarray analysis. Combined with our data on cellular stress, and the validation of our gene expression results through qRT-PCR and protein quantification, this study provides deeper insight into the molecular events that occur during exposure to PM2.5. Future experiments will be required to do a comparison of PM2.5 from different locations instead of just Beijing, and investigate the roles of genes with significant change in toxic effects of PM2.5 exposure.

## Supporting Information

S1 Fig ROS generation in 16HBE cells that treated with extracts from clean quartz filters. The cells were incubated with 5 μM DCFH-DA and then treated with extracts from clean quartz filters. ROS production was monitored with the fluorescence intensity by microscopic analysis. A. Changes in intracellular ROS levels before and after exposure. Time 0 means the start of a measurement. The arrow indicates the time at which control sample was added to culture media. The five lines show the DCF fluorescence intensity obtained from five arbitrary single cells in the same field of the microscope. B. Fluorescent localization of ROS. Scale bar in graphs, 20 μm.
(DOCX)
