# Supplementary material for: Transcriptomic Analyses of the Biological Effects of Airborne PM2.5 Exposure on Human Bronchial Epithelial Cells
Source: PLoS One. 2015 Sep 18;10(9):e0138267. doi: 10.1371/journal.pone.0138267 (PMC4575100; doi:10.1371/journal.pone.0138267)
Supplement: S1 Fig — The cells were incubated with 5 μM DCFH-DA and then treated with extracts from clean quartz filters. ROS production was monitored with the fluorescence intensity by microscopic analysis. A. Changes in intracellular ROS levels before and after exposure. Time 0 means the start of a measurement. The arrow indicates the time at which control sample was added to culture media. The five lines show the DCF fluorescence intensity obtained from five arbitrary single cells in the same field of the microscope. B. Fluorescent localization of ROS. Scale bar in graphs, 20 μm. (DOCX) [file pone.0138267.s001.docx]

Supporting Information

Fig. S1. ROS generation in 16HBE cells that treated with extracts from clean quartz filters. The cells were incubated with 5 μM DCFH-DA and then treated with extracts from clean quartz filters. ROS production was monitored with the fluorescence intensity by microscopic analysis. A. Changes in intracellular ROS levels before and after exposure. Time 0 means the start of a measurement. The arrow indicates the time at which control sample was added to culture media. The five lines show the DCF fluorescence intensity obtained from five arbitrary single cells in the same field of the microscope. B. Fluorescent localization of ROS. Scale bar in graphs, 20 μm.
